# Acute hyperglycaemia is not associated with the development of atrial fibrillation in healthy pigs

**DOI:** 10.1038/s41598-020-68897-0

**Published:** 2020-07-17

**Authors:** Martin Manninger, David Zweiker, Martin Dobrovnik, Arne van Hunnik, Ursula Rohrer, Birgit Zirngast, Viktoria Herbst, Heinrich Maechler, Ulrich Schotten, Andreas Zirlik, Daniel Scherr

**Affiliations:** 1 Department of Cardiology, Medical University of Graz, Auenbruggerplatz 15, 8036 Graz, Austria; 2 Department of Physiology, Cardiovascular Research Institute Maastricht, University of Maastricht, Universiteitssingel 50, Maastricht, The Netherlands; 3 Department of Cardiothoracic Surgery, Medical University of Graz, Auenbruggerplatz 29, 8036 Graz, Austria

**Keywords:** Arrhythmias, Diabetes

## Abstract

Development and progression of atrial fibrillation (AF) is driven by comorbidities such as arterial hypertension and diabetes mellitus. In animal models of chronic hyperglycaemia, progression of AF has been proposed to be triggered by oxidative stress, apoptosis and fibrosis. Acute glycosylation of CaMKII has been associated with increased susceptibility to arrhythmias in acute hyperglycaemia. However, the proarrhythmogenic effect of acute hyperglycaemia has not been investigated. Nine healthy, anesthetized pigs (54 ± 6 kg) were instrumented with electrophysiologic catheters and a multielectrode array on the epicardium of the left atrial anterior wall. Left and right atrial effective refractory periods (AERP), inducibility of AF and left atrial epicardial conduction velocities (CV) were measured at baseline (BL), increasing steps of blood glucose (200–500 mg/dL in steps of 100 mg/dL by glucose infusion) and repeated after normalisation of blood glucose levels (recovery). Serum electrolytes were kept constant during measurements by means of sodium and potassium infusion. There were no significant differences in AERP, CV or AF inducibility between BL and recovery. Heart rate remained constant regardless of blood glucose levels (BL: 103 ± 18 bpm, 500 mg/dL: 103 ± 18 bpm, r = 0.02, p = 0.346). Mean left as well as right AERP increased with higher glucose levels. CV increased with glucose levels (1.25 (1.04, 1.67) m/s at BL vs. 1.53 (1.22, 2.15) m/s at 500 mg/dL, r = 0.85, p = 0.034). Rate of AF inducibility in the left atrium remained constant throughout the whole protocol (AF episodes > 10 s: mean inducibility of 80% at BL vs. 69% at 500 mg/dL, p = 0.32, episodes > 30 s: 0% at BL vs. 0% at 500 mg/dL, p = 0.17). Our data imply that acute hyperglycaemia is associated with lower arrhythmogenic substrate and does not promote AF inducibility.

## Introduction

Atrial fibrillation (AF) is the most common sustained arrhythmia in humans and is associated with an increased risk of stroke, morbidity and death. Progression of the disease is associated with risk factors such as structural heart disease, valvular heart disease, arterial hypertension, diabetes mellitus (DM) and obesity^1–3^. Extensive research is conducted on how exactly these conditions favour the development and progression of AF, but the exact mechanisms remain unknown^4^.

In early stages of AF, triggers like ectopic beats from the pulmonary veins, atrial premature beats or episodes of atrial tachycardia initiate the arrhythmia. Recurrent episodes of AF lead to electrical remodelling of the atria, including shortening of atrial effective refractory periods action potential duration and a loss of rate adaption which favour arrhythmia stability (“AF begets AF”)^4,5^. During the development to persistent AF, the substrate (i.e. changes in atrial architecture, alteration of extracellular matrix composition, dissociation of endo- and epicardium) gains in importance over the initiating triggers^4,6^.

Fifteen percent of patients with AF suffer from DM^7^. Patients with impaired fasting glucose or DM have an elevated risk (33% per mmol/L increased fasting glucose) of developing AF^8,9^.

Prior animal studies have suggested that oxidative stress, apoptosis and inflammation favour AF progression in the presence of DM^10^. Glycosylation of CaMK II (calcium/calmodulin-dependent protein kinase II) has been proposed as a possible proarrhythmic mechanism in DM or hyperglycaemia^11^. Glycosylation of CaMK II favours afterdepolarisations, one of the key mechanisms in AF development^4^. This mechanism has been demonstrated in explanted human ventricular myocardium, in diabetic rats and in cell cultures^11^.

However, it is not known, if acute hyperglycaemia affects the atrial electrical substrate and increases AF inducibility. In the present study, we investigated, whether the effect of acute hyperglycaemia on the electrical substrate is a possible determinant for AF inducibility and stability.

## Materials and methods

The experimental protocol was approved by the local bioethics committee of Vienna, Austria (BMWFW-66.010/0100-WF/V/3b/2016), and conforms to the “European Convention for the Protection of Vertebrate Animals used for Experimental and other Scientific Purposes” (Council of Europe No 123, Strasbourg 1985).

### Experimental setup

The experimental setup has been described before^12–14^. Briefly, landrace pigs (n = 9, 54 ± 6 kg) were fasted overnight, premedicated with midazolam and ketamine, intubated and mechanically ventilated. Anaesthesia was continued with sevoflurane, fentanyl, midazolam, pancouronium and ketamine.

Sheaths were introduced into both carotid arteries, internal jugular veins and femoral veins. A quadripolar stimulation catheter was positioned in the high right atrium (Response 6F, St. Jude Medical, Saint Paul, MN, USA), a decapolar reference catheter (6 F Dynamic Tip Steerable Catheter, Bard Electrophysiology, Lowell, MA, USA) in the coronary sinus and a steerable sheath (Agilis, St. Jude Medical, Lowell, MN, USA) with a quadripolar mapping catheter (Thermocool, Biosense Webster, Johnson & Johnson, Irvine, CA, USA) first in the right atrium and subsequently in the left atrium after transseptal puncture. Pacing was performed over the quadripolar stimulation catheter in the RA and the quadripolar mapping catheter in the LA using an external stimulator (UHS20, Biotronik, Germany).

### Electrophysiologic study

Atrial effective refractory period (AERP) was determined using a S1–S2 stimulation protocol (1 ms pulse at twice diastolic threshold at cycle lengths 400 ms, 350 ms, 300 ms, 250 ms and 200 ms) as described before^14^.

Inducibility of AF was assessed by burst protocols (1 ms pulse at four times diastolic threshold, cycle length 20 ms, 10 s duration, five repetitions). An AF episode was defined as the post-burst onset of irregular electrograms with an average atrial cycle length ≤ 200 ms and ≥ 10 s of duration.

### Epicardial multielectrode mapping

To allow contact mapping of the left atrium, median thoracotomy was performed. The pericardium was opened for 2–3 cm to place a custom-made, squared high-density mapping electrode array (16 × 16 channels, 1.5 mm interelectrode distance) on the left atrial free wall. To measure conduction velocities, pacing at cycle lengths from 400 to 200 ms with decrements of 50 ms was performed in the proximal coronary sinus. Electrograms were recorded for 30 s (sampling rate 1 kHz, filtering bandwidth 0.5–500 Hz).

Local deflections in each recorded electrogram were identified using a probabilistic electrogram algorithm. For each activation at each electrode, a plane was fitted to activation times at neighbouring electrodes (maximum square of 3 × 3) belonging to the same wave. The plane indicates local direction of propagation and conduction velocity^15,16^. Conduction velocity was analysed using custom-made software^17^.

### Induction of hyperglycaemia

Measurements were started at fasting glucose levels (BL—baseline) and repeated at glucose levels of 200, 300, 400 and 500 mg/dL. Glucose levels were elevated by means of glucose infusion (Glucose 5%, Fresenius Kabi, Austria), serum electrolytes were kept constant during measurements by means of sodium and potassium infusion (NaCl 0.9% and “Elozell spezial”, Fresenius Kabi, Austria). Then, glucose infusions were stopped, and measurements were repeated after glucose levels returned to the level of the first BL measurements (recovery). Animals were allowed to stabilize for 15 min at each glucose step before measurements were repeated.

### Blood samples

At each step of the experimental protocol, arterial blood samples were taken and glucose levels, electrolytes, oxygen saturation, partial oxygen and carbon dioxide pressures, pH, acid–base status, haemoglobin and lactate were measured using a blood gas analyser (ABL 600; Radiometer, Copenhagen, Denmark).

### Data processing and statistical analyses

Categorical variables are presented as percentages (%) and counts, continuous variables as mean ± SD or median (range). Two-group comparisons of normally distributed continuous variables were performed by the Student’s t test. In case normality assumption was violated according to Shapiro–Wilk tests, the Wilcoxon rank-sum test was used. Categorical variables were compared using the chi-square test. EP data at different glucose levels, at different pacing steps or pacing cycle lengths were compared by 1- or 2-way repeated measurement analysis of variance, Tukey’s test was used for post-hoc analysis. Two-tailed P values < 0.05 were considered statistically significant. Correlations were calculated using Pearson's correlation coefficient. Statistical analyses were performed using SPSS 20.0 (IBM, Armonk, NY, USA), graphs were plotted with Prism 6 (GraphPad Software Inc., California, USA).

## Results

All nine animals were included in the final analysis. There were no significant differences between measurements during BL and recovery, so results from recovery phase will not be reported here. Mean glucose level at BL was 92 ± 18 mg/dL.

Heart rate remained constant regardless of blood glucose levels (BL: 103 ± 18 bpm, 200 mg/dL: 98 ± 17 bpm, 300 mg/dL: 98 ± 16 bpm, 400 mg/dL: 99 ± 14 bpm, 500 mg/dL: 103 ± 18 bpm, r = 0.02, p = 0.346).

### Electrophysiologic study

**Figure 1:**
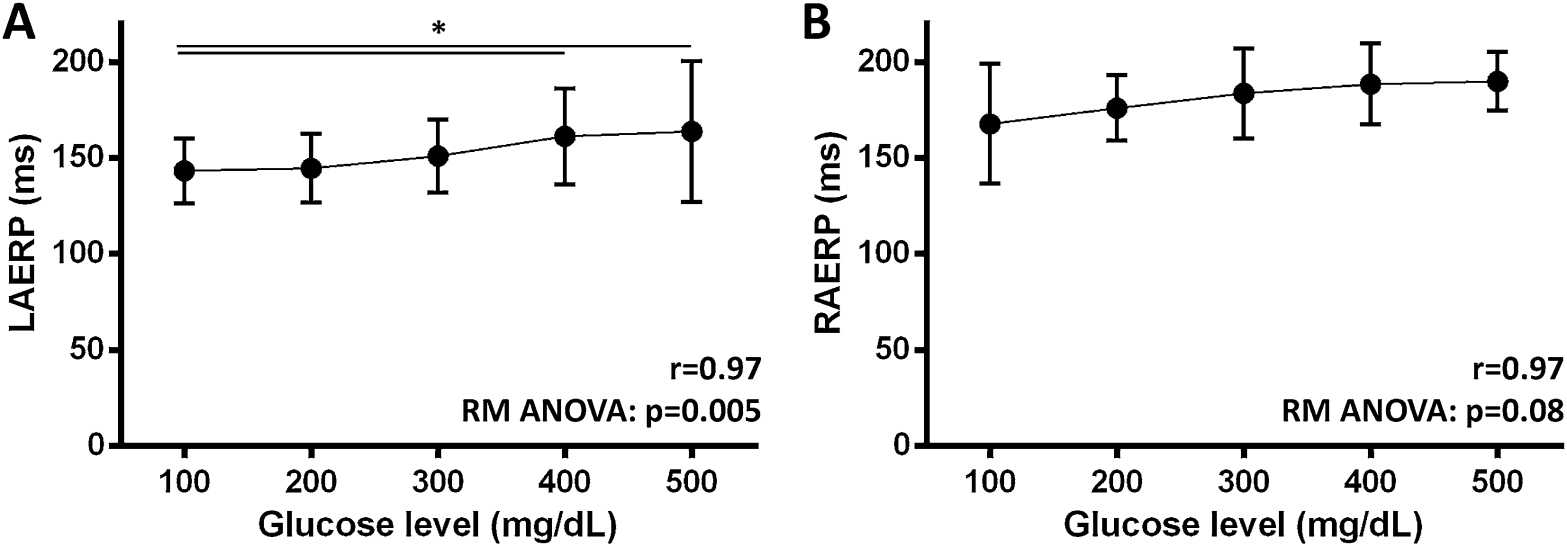
Left (**A**) and right (**B**) atrial effective refractory periods with increasing glucose levels (*p < 0.05 in post-hoc analysis, error bars indicate standard deviation).

**Figure 2:**
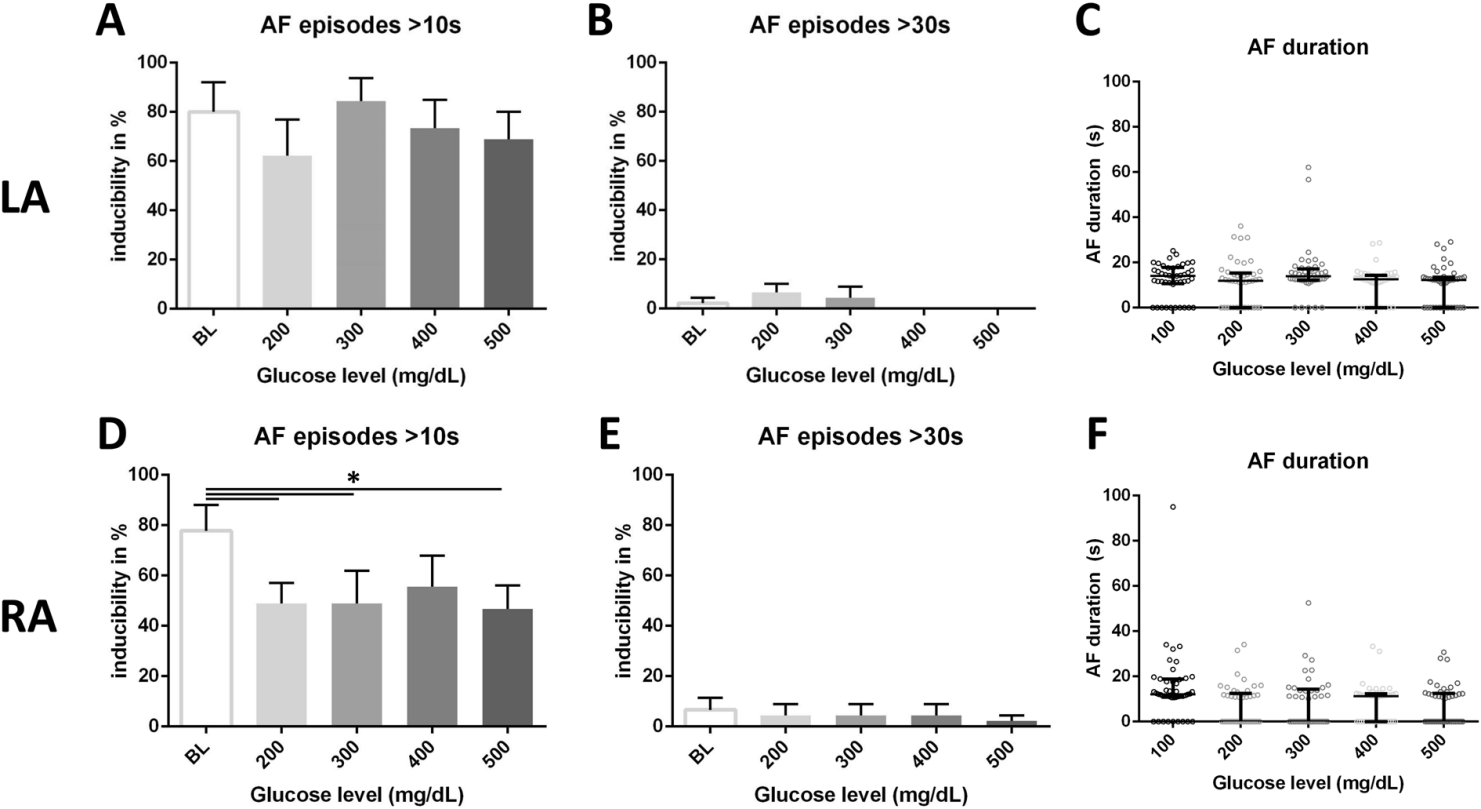
Left atrium: Mean incidence of AF longer than 10 s (**A**), longer than 30 s (**B**) and median AF duration (**C**) after atrial burst pacing with increase in blood glucose levels. Right atrium: Mean incidence of AF longer than 10 s (**D**), longer than 30 s (**E**) and median AF duration (**F**) after atrial burst pacing with increase in blood glucose levels (*p < 0.05 in post-hoc analysis, error bars indicate SEM/IQR).

Mean left (r = 0.97, p = 0.003, Fig. 1A) as well as right AERP (r = 0.97, p = 0.003, Fig. 1B) increased with higher glucose levels. In stepwise comparison, left AERP at 400 and 500 mg/dL was higher compared to baseline (Fig. 1A, p = 0.005), while there was a similar trend in right AERP (p = 0.08). AF inducibility was not increased during hyperglycaemia. In the left atrium, AF episodes longer than 10 s were inducible in 80% at BL, 62% at 200 mg/dL, 84% at 300 mg/dL, 73% at 400 mg/dL, 69% at 500 mg/dL (p = 0.32, Fig. 2A). AF episodes longer than 30 s were inducible only at two drive trains in one animal at 200 mg/dL (p = 0.17, Fig. 2B). In the right atrium, AF episodes longer than 10 s were inducible in 78% at BL, 49% at 200 mg/dL, 49% at 300 mg/dL, 56% at 400 mg/dL, 47% at 500 mg/dL (p = 0.016, Fig. 2D). Compared to BL, short AF episodes were significantly less induced at 200 (p = 0.038), 300 (p = 0.038) and 500 mg% (p = 0.022). AF episodes longer than 30 s were inducible only at one drive train in one animal at BL (p = 0.94, Fig. 2E). Median AF duration did not differ between all glucose steps in the left (p = 0.23, Fig. 2C) and right (p = 0.12, Fig. 2F) atrium.

No ectopic activity was observed during sinus rhythm to the highest pacing rates.

### Epicardial conduction velocity

**Figure 3:**
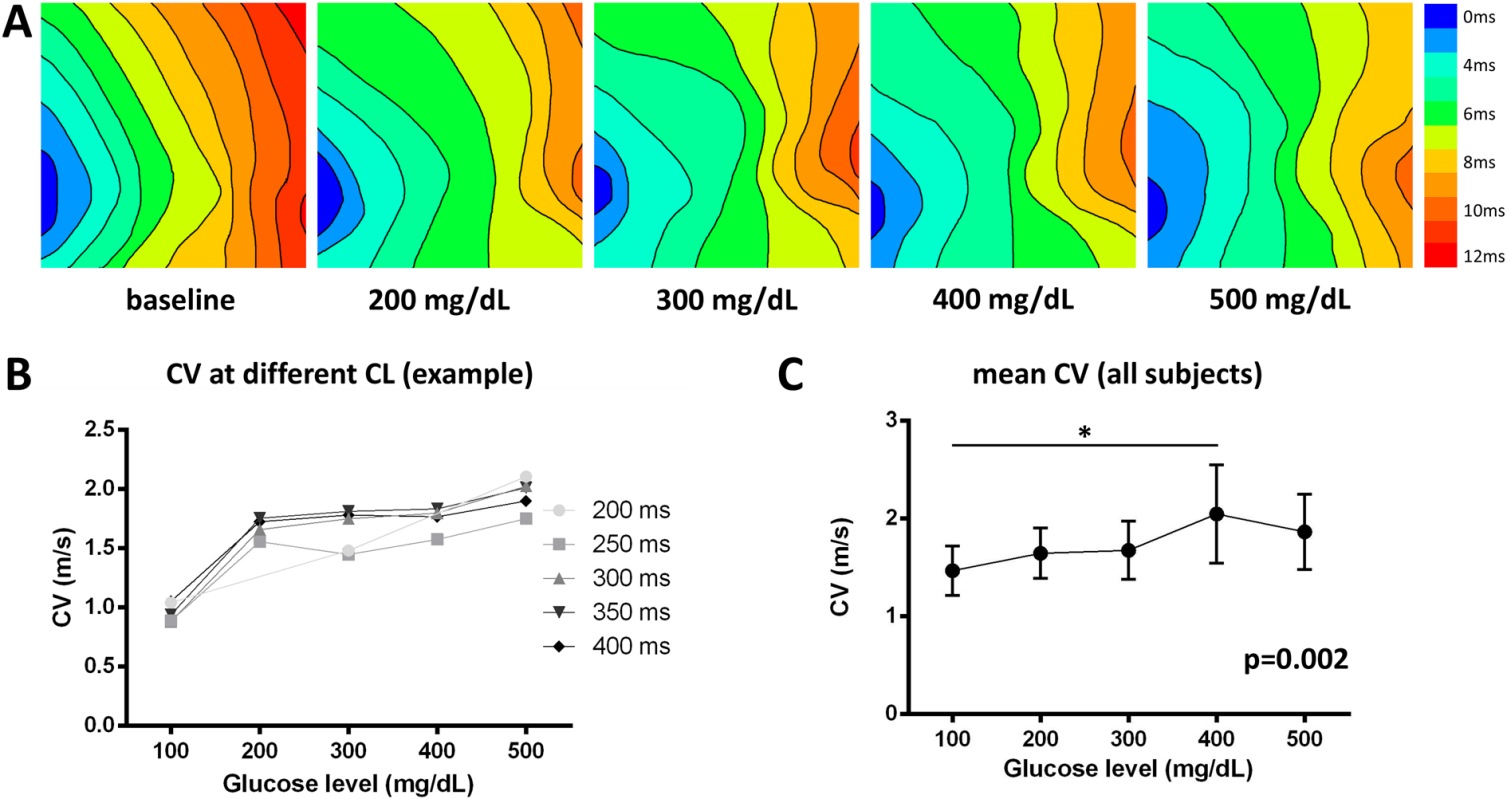
**A** Representative propagation maps at an S1 pacing cycle length of 400 ms with isochrones of 2 ms (earlies activation in blue, latest in red) of the left atrium. **B** Representative conduction velocities of the same animal at different pacing cycle lengths (S1 400–200 ms in decrements of 50 ms). **C** Mean conduction velocities increased with increasing glucose levels (*p < 0.05 in post-hoc analysis, error bars indicate SEM).

Mean CV increased with glucose levels (1.25 (1.04, 1.67) m/s at BL, 1.39 (1.17, 1.94) m/s at 200 mg%, 1.37 (1.12, 2.01) m/s at 300 mg%, 1.73 (1.24, 2.17) m/s at 400 mg% and 1.53 (1.22, 2.15) m/s at 500 mg%, r = 0.85, p = 0.034, Fig. 3). Mean CV at 400 mg% was higher compared to BL (p = 0.002).

### Serum electrolytes

Although electrolytes were substituted and kept within a normal range, exact balance throughout the protocol was not achieved (Table 1). Serum potassium (BL: 3.5 ± 0.15 mmol/L, 500 mg%: 3.84 ± 0.43; r = 0.92, p = 0.005) was over substituted, while sodium decreased with increasing glucose (BL: 140 (139.5, 141.5) mmol/L, 500 mg%: 132 (129, 136.5) mmol/L, r = 0.99, p < 0.001). Serum calcium and chloride levels remained unchanged. pH decreased with increasing glucose levels.

**Table 1:** Serum glucose, serum electrolyte levels and heart rate during repeated measurements.

	BL	200 mg/dL	300 mg/dL	400 mg/dL	500 mg/dL	p-value
Glucose (mg/dL)	92 ± 18	242 ± 23	305 ± 35	408 ± 24	512 ± 20	
Na (mmol/L)	140 (139.5, 141.5)	138 (136.5, 139)	136 (134, 137)	134 (130.5, 137)	132 (129, 136.5)	< 0.001
K (mmol/L)	3.5 ± 0.2	3.5 ± 0.3	3.8 ± 0.3	3.8 ± 0.3	3.8 ± 0.4	0.005
Ca (mmol/L)	1.15 (1.12, 1.215)	1.1 (1.04, 1.24)	1.18 (1.13, 1.26)	1.22 (1.095, 1.03)	1.12 (1.09, 1.26)	0.171
Cl (mmol/L)	103 (101, 104)	99 (98, 101.5)	99 (96.5, 102)	97 (94, 104.5)	96 (04.5, 105)	0.16
pH	7.48 ± 0.03	7.46 ± 0.02	7.44 ± 0.03	7.40 ± 0.03	7.36 ± 0.02	< 0.001
Heart rate (/min)	103 ± 18	98 ± 17	98 ± 16	99 ± 14	103 ± 18	0.346

## Discussion

Patients with DM are more likely to develop AF and DM favours the progression of AF^7–10,18^. Animal studies have suggested that chronic factors e.g. oxidative stress, apoptosis and inflammation play and favour AF progression in the presence of DM^10^. However, it is not known, if hyperglycaemia per se represents an acute arrhythmogenic substrate or if AF develops secondarily to structural atrial changes. Here, we could demonstrate that susceptibility for AF does not change due to acute hyperglycaemia. Interestingly, acute hyperglycaemia was more associated with a strong increase in pathlength and shorter AF episodes.

### Electrophysiological effects of hyperglycemia

We could demonstrate in our study, that hyperglycemia leads to prolongation of CV, ERP resulting in shorter AF durations.

The only in vivo study on the role of glucose levels on atrial electrophysiology was performed in open chest dogs^19^. Inducibility of AF was higher in hypoglycaemia than in normo- and hyperglycaemia, while refractory periods tended to increase with the glucose levels. This could be explained by the fact that animals were in hypokalaemia during hypoglycaemia. Notably, glucose levels showed a large variability and tests were performed at only one level of hyperglycaemia at a median glucose level of 280 mg%.

Erickson et al. demonstrated that hyperglycaemia leads to glycosylation of CaMKII^11^. Acute glucose elevation activates CaMKII which leads to spontaneous sarcoplasmic reticulum calcium release events that can contribute to development of arrhythmias. This could be demonstrated in intact hearts and diabetic rats, where glucose-induced premature ventricular complexes and ventricular tachycardias could be suppressed by blockade of CaMKII or glycosylation of CaMKII. It remains unclear, if this mechanism might also play a role in pathogenesis of AF in patients with DM. Since AF inducibility was not increased in this large animal study when electrolytes were kept within a normal range, longer durations of hyperglycaemia might be necessary for this mechanism to become relevant.

Shortening of atrial refractory periods is attributed to an increased stability of AF, which is described as electrical remodelling during the progression of atrial fibrillation^5,20^. Henceforward, therapeutic agents prolonging APD and refractory periods are used for cardioversion of patients^4^. In the setting of hyperglycaemia, prolonged refractory periods seem to protect the atria from sustaining AF induced by burst pacing. Our findings that hyperglycaemia prolongs refractory periods are in line with prior in vivo and in vitro studies^19,21^.

In other animal studies, increased AF stability was often associated with a reduction in conduction velocity^15,22–24^. In our study, conduction velocities were increased by hyperglycaemia, which may also contribute to the unchanged AF inducibility throughout the protocol. It may also explain the fact that with the right atrium, short AF episodes were even less frequent with increasing glucose levels.

### Effect of DM on the development and progression of AF

Clinical studies have shown that impaired glucose tolerance and DM are associated with an increased risk of developing AF in all-comers^8,9,18^, patients after coronary artery bypass graft surgery^25^ and after myocardial infarction^26^. In patients undergoing AF ablation, impaired glucose tolerance and DM are associated with structural remodelling (i.e. longer total atrial conduction times, lower bipolar voltages)^18^. These findings suggest that DM promotes AF through structural alterations.

The role of DM in structural atrial remodelling has been studied in multiple animal models. In mice with stroptozoticin-induced hyperglycaemia, it has been suggested that mast cells contribute to the pathogenesis of DM-induced AF via enhancement of inflammation and fibrosis^27^. Genetic type II diabetic rats showed increased atrial arrhythmogenicity, intra-atrial conduction disturbances attributed to interstitial fibrosis, while refractory periods were comparable to wild-type rats^28^. In Langendorff-perfused hearts of rabbits with alloxan-induced DM, AF inducibility was increased while refractory periods and action potential durations were prolonged. These changes were also attributed to structural alterations (i.e. increase in collagen volume fraction)^21^. In Langendorff-perfused hearts of rats with streptozoticin-induced DM, AF inducibility and heterogeneity of refractory periods were increased under sympathetic nerve stimulation, suggesting that neural remodelling might also play a role the pathogenesis of AF in the presence of DM^29^.

Our findings suggest that rather systemic and structural changes during DM than acute hyperglycaemia play an important role in AF pathogenesis. This is in line with prior animal studies that require longer exposure to a diabetic state in order to manifest increased susceptibility to AF.

## Limitations

Over-substitution of potassium might affect inducibility of AF, however, electrolytes were kept within a normal range throughout the whole protocol.

We performed our experiments in healthy pigs without cardiac disease. We may not exclude that arrhythmogenic properties increase in the context of structurally remodeled atria. However, we do not have evidence that a more pathophysiological behavior occurs in remodeled atria. Moreover, the increased CV suggest an increased safety factor for conduction leading to a lower likelihood of wave break and complex conduction. Therefore, we anticipate that our findings can be translated to clinical scenarios where DM is already present. However, a new series of experiments would be necessary to verify this notion.

Since induced AF episodes were short and unstable, we could not perform further qualitative analyses on AF patterns.

## Conclusion

Acute hyperglycaemia does not represent a substrate for increased AF inducibility in a large animal model.
